# Early detection of neutralizing antibodies against SARS-CoV-2 in COVID-19 patients in Thailand

**DOI:** 10.1371/journal.pone.0246864

**Published:** 2021-02-12

**Authors:** Opass Putcharoen, Supaporn Wacharapluesadee, Wan Ni Chia, Leilani Paitoonpong, Chee Wah Tan, Gompol Suwanpimolkul, Watsamon Jantarabenjakul, Chanida Ruchisrisarod, Phanni Wanthong, Jiratchaya Sophonphan, Pajaree Chariyavilaskul, Lin-Fa Wang, Thiravat Hemachudha

**Affiliations:** 1 Division of Infectious Diseases, Department of Medicine, Faculty of Medicine, Thai Red Cross Emerging Infectious Diseases Clinical Centre, King Chulalongkorn Memorial Hospital, Chulalongkorn University, Bangkok, Thailand; 2 Thai Red Cross Emerging Infectious Diseases Health Science Centre World Health Organization Collaborating Centre for Research and Training on Viral Zoonoses, King Chulalongkorn Memorial Hospital, Faculty of Medicine, Chulalongkorn University, Bangkok, Thailand; 3 Programme in Emerging Infectious Diseases, Duke-NUS Medical School, Singapore, Singapore; 4 Division of Infectious Diseases, Department of Pediatrics, Faculty of Medicine, Thai Red Cross Emerging Infectious Diseases Clinical Centre, King Chulalongkorn Memorial Hospital, Chulalongkorn University, Bangkok, Thailand; 5 The HIV Netherlands Australia Thailand Research Collaboration (HIV-NAT), Bangkok, Thailand; 6 Clinical Pharmacokinetics and Pharmacogenomics Research Unit, Faculty of Medicine, Chulalongkorn University, Bangkok, Thailand; 7 Department of Pharmacology, Faculty of Medicine, Chulalongkorn University, Bangkok, Thailand; University of Hong Kong, HONG KONG

## Abstract

**Background:**

The presence of neutralizing antibodies (NAbs) is an indicator of protective immunity for most viral infections. A newly developed surrogate viral neutralization assay (sVNT) offers the ability to detect total receptor binding domain-targeting NAbs in an isotype-independent manner, increasing the test sensitivity. Thus, specimens with low IgM/ IgG antibody levels showed strong neutralization activity in sVNT.

**Methods:**

This study aimed to measure the %inhibition of NAbs measured by sVNT in PCR-confirmed COVID-19 patients. The sensitivity of sVNT for the diagnosis of SARS-CoV-2 infection and its kinetics were determined.

**Results:**

Ninety-seven patients with PCR-confirmed SARS-CoV-2 infection were included in this study. Majority of the patients were 21–40 years old (67%) and 63% had mild symptoms. The sensitivity of sVNT for the diagnosis of SARS-CoV-2 infection was 99% (95% confidence interval (CI) 94.4–100%) and the specificity was 100% (95% CI 98.3–100%). The negative predictive value of sVNT from the samples collected before and after 7 days of symptom onset was 99.5% (95% CI 97.4–100%) and 100% (95% CI 93.8–100%), respectively. The level of inhibition at days 8–14 were significantly higher than days 0–7 (p<0.001). The median %inhibition values by severity of COVID-19 symptoms were 79.9% (interquartile range (IQR) 49.7–91.8%); 89.0% (IQR 71.2–92.4%); and 86.6% (IQR 69.5–92.8%), for mild, moderate and severe/critical symptoms respectively. The median level of sVNT %inhibition of severe was significantly higher than the mild group (p = 0.05).

**Conclusion:**

The sVNT is a practical and robust serological test for SARS-CoV-2 infection and does not require specialized biosafety containment. It can be used clinically to aid diagnosis in both early and late infection especially in cases when the real-time RT-PCR results in weakly negative or weakly positive, and to determine the protective immune response from SARS-CoV-2 infection in patients.

## Introduction

Thailand’s population is approximately 70 million with approximately 10% of the country’s population residing in its capital city, Bangkok, and its metropolitan areas. As of 31 May 2020, Thailand’s confirmed cases count was 3081, of which 1515 (49%) was reported within Bangkok. Since initial reports of COVID-19, Thailand has implemented several measures to reduce its spread. Among the innumerable challenges presented by the Coronavirus Disease 2019 (COVID-19) pandemic worldwide [1], diagnostic testing has been among the most pressing, and has been central to limiting the spread of severe acute respiratory syndrome coronavirus 2 (SARS-CoV-2) infection. Reverse transcriptase polymerase chain reaction (RT-PCR), to detect the viral nucleic acid directly from clinical specimens, is the standard diagnostic method and is recommended by the US CDC [2]. A robust and reliable serological test for antibodies against the virus, however, is greatly needed to enable functions for which RT-PCR is not suitable such as retrospective contact tracing, case fatality determinations, rapid and inexpensive diagnosis of asymptomatic infection, seroprevalence assessments, humoral immunity assessment to be used in screening for potential convalescent plasma donors [3], and monitoring immune response in vaccine candidates [4, 5].

Neutralizing antibodies are specific for viral surface epitopes that mediate entry of the virus into a host cell [6]. In SARS-CoV-2, these epitopes are predominantly located in the receptor binding domain (RBD) of the spike (S) protein [7]. The presence of neutralizing antibodies are a good indicator of protective immunity for most viral infections and it is reasonable to assume that this is also true for SARS-CoV-2 infection, as demonstrated by a recent study [8]. A serological assay capable of directly measuring neutralizing antibodies would thus be preferable to those serological tests which assay for binding antibodies. The current reference standard for detecting neutralizing antibodies is the virus neutralization test (VNT), which can be a plaque reduction neutralization test (PRNT), pseudotype virus neutralization assay or a microneutralization test. However, both tests require the use of live viruses and a biosafety level 3 containment facility, and thus are too cumbersome to be routinely performed [5].

To address this problem, a recently developed assay makes use of the pathobiological affinity between the RBD of the S protein and the angiotensin converting enzyme 2 (ACE2) receptor proteins which are typically found on target host cells [9]. When expressed in the correct conformation and in a soluble form, purified RBD and ACE2 can interact with each other *in vitro* with sufficient affinity for easy monitoring. Neutralizing antibodies against RBD compete with ACE2 to bind RBD, and this is the basis for the clinical assay to detect these specific antibodies. The degree to which a patient’s serum or plasma inhibits the RBD-ACE2 interaction can be measured by labelling the purified proteins colorimetrically and the degree of inhibition correlates with the quantity of neutralizing antibodies. The result is a surrogate virus neutralization test (sVNT) to detect neutralizing antibodies (NAbs) against SARS-CoV-2, where the RBD acts as the surrogate of the virus and ACE2 as the surrogate of the susceptible cell. This assay has been tested previously in two large cohorts in two Asian countries, and is commercially available (under the trade name cPass^TM^, GenScript, USA). The sVNT offers the ability to detect total RBD-targeting NAbs in an isotype-independent manner, thereby increasing the test sensitivity [9].

### Objectives

In this study, we aimed to measure sVNT levels (%inhibition) in PCR-confirmed COVID-19 patients. The levels and kinetics of neutralizing antibodies (NAbs) were compared with patients’ clinical and demographic data. In addition, a comparison between the sensitivity and specificity of sVNT and a commercial IgA/IgG immunoassay was performed.

## Methods

The study was conducted at the Thai Red Cross Emerging Infectious Diseases (TRC-EID) Clinical Centre (TRC-EID-CC) and Health Science Centre (TRC-EID-HSC), King Chulalongkorn Memorial Hospital, Faculty of Medicine, Chulalongkorn University. The study was reviewed and approved by the Institutional Review Board at the Faculty of Medicine, Chulalongkorn University (IRB number 400/63). Written informed consent was obtained from all individual participants.

In the overall design of this study, blood samples from PCR-confirmed COVID-19 patients underwent testing for NAbs against SARS-CoV-2. The correlation between the level of inhibition by sVNT and clinical parameters were determined.

### Patient selection and samples

We included the patients who presented to the TRC-EID-CC between January 15, 2020 and April 5, 2020 with confirmed COVID-19 by rRT-PCR. All patients were tested for SARS-CoV-2 infection if they met the PUI (Patient Under Investigation) criteria which included a history of exposure to patients with COVID-19 or a history of travel to COVID-affected areas within the past 2 weeks, and/or clinical symptoms as defined by the Thai Ministry of Public Health such as fever, myalgia, rhinorrhea, cough, and loss of sense of smell. Nasopharyngeal and throat (NT) swabs were collected from all PUIs and tested for SARS-CoV-2 using standard real-time RT-PCR (rRT-PCR). Additional blood samples were collected for serological testing and patients were included in the current study if at least 2 blood samples were collected and available.

Patients with PCR-confirmed COVID-19 were classified by disease severity, as follows: 1) mild or upper respiratory tract infection; 2) moderate–pneumonia without oxygen desaturation treated with antiviral agents for 5 days or other combination regimens; 3) severe–pneumonia treated with oxygen support and antiviral agents for at least 10 days; 4) critical–pneumonia requiring mechanical ventilation. These severity grades were analogous to the WHO blueprint grades 3, 4 and 5 respectively. All patients were admitted to TRC-EID-CC, King Chulalongkorn Memorial Hospital, and most underwent serial rRT-PCR and serology testing for following up. All patient’s NT specimens were periodically tested for SARS-CoV-2 using rRT-PCR until the result turned negative before discharging.

Control-negative serum samples for serological testing were obtained from the National Blood Centre, Thai Red Cross Society, and from COVID-19 PCR negative patients who presented to the TRC-EID. A total of 298 pre-pandemic sera from blood bank archive for negative control and test for specificity of sVNT. These samples were collected before 2020.

### Real-time reverse transcription polymerase chain reaction (rRT-PCR)

NT swabs from all suspected COVID-19 patients underwent rRT-PCR testing for SARS-CoV-2 at the TRC-EID-HSC or at the microbiology laboratory of the Faculty of Medicine, Chulalongkorn University. At TRC-EID-HSC Nucleic acid (NA) extraction was performed on all samples using magLEAD kit according to the manufacturer’s instructions (Precision System Science) and the RT-qPCR was performed using Seegene—Allplex 2019-nCov Assay, in a Bio-Rad CFX 96 qPCR machine according to manufacturer’s instruction. Briefly, reaction was heated to 50oC for 20 minutes for reverse transcription, denatured in 95oC for 15 minutes and then 45 cycles of amplification was carried in 94oC for 15 seconds and 58oC for 30 seconds. Fluorescence was measured using four fluorescence channels: FAM (E gene), HEX (internal control), Cal Red 610 (RdRp gene), Quasar 670 (N gene). The protocol’s stated limit of detection of ORF1ab rRT-PCR was 1000 copies/mL and the cut-off PCR cycle threshold (Ct) was 40.

At the microbiology laboratory the cobas® 6800 system (Roche Diagnostics, Switzerland) was used for detection of SARS-CoV-2 RNA which targeting conserved regions within the ORF 1a/b and E genes, according to manufacturer’s instruction.

### Binding antibodies (BAbs) tests

A commercial, semi-quantitative assay for detecting immunoglobulin classes IgA and IgG (EUROIMMUN, Germany) was used for antibody detection of SARS-CoV-2 infection. The kit contains microplate strips of 8 break-off reagent wells coated with recombinant structural proteins of SARS-CoV-2. In the first reaction, diluted patient’s samples were incubated in the wells. After rinsing off unbound antibodies, those remaining were detected using colorimetrically labelled antihuman IgA and IgG. The optical density (OD) at 450 nm was recorded and a ratio of each sample reading to the reading of the calibrator was calculated. The results were semiquantitative and were reported as a ratio compared with controls: < 0.8 negative; ≥ 0.8 to 1.0 borderline; ≥ 1.0 positive. Borderline results were considered as positive. In addition, to compare sensitivity and specificity of serologic tests for COVID-19, IgM and IgG against RBD region of SARS-CoV-2 region was tested in each sample.

### Neutralizing antibodies (NAbs) tests

Blood specimens from patients with PCR-confirmed COVID-19 were tested for NAbs against SARS-CoV-2 antibodies using sVNT assay (cPass™, GenScript USA), according to the manufacturer’s instructions. Briefly, horseradish peroxidase (HRP)–RBD was pre-incubated with test serum (1:10 diluted) for 1 hour at 37°C after which it was added onto the ELISA plate pre-coated with hACE2 (GenScript). The unbound HRP-RBD was washed off, and bound RBD-ACE2 was detected colorimetrically. Circulating NAbs against SARS-CoV-2 competitively inhibited the RBD-ACE2 interaction. The percentage of inhibition was calculated by measuring the difference in the amount of labelled RBD between test versus control samples. The cutoff ratio for percentage of inhibition was at 20%. In addition, a set of samples with known levels of SARS-CoV-2 by conventional virus neutralization test (cVNT) were used to evaluate the correlation between this assay and sVNT. Two-fold dilution from 1:10 to 1:320 was performed to determine the titer.

### Conventional virus neutralization test (cVNT)

Briefly, the sample or positive control or negative control was twofold serially diluted in 96-well plates. Then, 100 × TCID50 (50% tissue culture infectious dose) of the SARS-CoV-2 virus was added to the mAb dilutions and incubated for 1 h at 37°C. The Vero-E6 cells pre-seeded in Dulbecco’s modified Eagle’s medium (DMEM) supplemented with 2% fetal bovine serum (FBS), 100 U/ml of penicillin, and 0.1 mg/ml of streptomycin (1 × 104 cells/well, duplicates) were then infected with the mAb–virus mixture for 2 days. The cells were then fixed and permeabilized with ice-cold 1:1 methanol/acetone fixative for 20 min at 4°C. The fixed plates were washed three times with 1 × PBS containing 0.05% Tween 20 (wash buffer) and blocked with blocking buffer containing 2% bovine serum albumin (BSA) and 0.1% tween 20 in 1 × PBS for 1 h. After washing the plates thrice with wash buffer, the SARS-CoV/SARS-CoV-2 nucleocapsid mAb (40143-R001; Sino Biological, United States) with a dilution of 1:5,000 in 1 × PBS containing 0.5% BSA and 0.1% Tween 20 was added to each well and incubated for 2 h at 37°C. The plates were then washed three times with wash buffer, and HRP-conjugated goat anti-rabbit polyclonal antibody (P0448; Dako, Denmark) was used as secondary antibody at a dilution 1:2,000. After incubation for 1 h at 37°C, the plates were washed thrice and detected with SureBlue TMB 1 component microwell peroxidase substrate (SeraCare Life Sciences, United States). The reaction was stopped with 1N HCl. The absorbance was measured at 450 and 620 nm (reference wavelength) with an ELISA plate reader. Both virus control (no antibody) and cell control (no virus, no antibody) were included in the plates. Convalescent serum collected from the COVID-19 patient (heat inactivated at 56°C, 30 min) was used as a positive control; anti-human PD1 antibody and negative serum were used as negative controls. The experiment was performed with three technical replicates for each sample. The average ODs at 450 and 620 nm were determined for virus control and cell control wells, and the neutralizing endpoint was determined by 50% specific signal calculation. Sera which tested negative at 1:10 dilution were assigned a titer of <10. Sera were considered positive if neutralization titer is >20.

## Statistical analysis

Demographic, clinical and laboratory data were collected for all patients. Continuous variables were expressed as median (interquartile range: IQR). Differences in continuous and categorical variables between the four clinical severity classification groups were assessed using Kruskal-Wallis test and Chi-squared test or Fisher’s exact test respectively. Logistic regression was used to determine factors associated with severe or critical patients. Multivariable models were developed by adjusting for covariates with p<0.1 in univariable models. All p-values reported were two-sided. Statistical significance was defined as p<0.05. Stata version 15.1 (Stata Corp., College Station, Texas), was used for analysis.

## Results

### Patient characteristics and demographic data

During the 3-month study period, 7,816 patients were screened at TRC-EID-CC. Of these, 825 met the criteria for PUI, and 158 (19.2%) were confirmed to have SARS-CoV-2 infection. Fifty-eight percent were female. The median age of confirmed cases was 35 (IQR 26–47) years. The majority (67%) of patients were aged 21–40 years. Of the 158 PCR confirmed cases, 63% were mild, 19% were moderate, 12% were severe, and 6% were critical. The median age of patients in the mild, moderate, severe and critical were 29 (IQR 24–38), 40 (33–50), 48 (38–58), and 54 (48–59) years old, respectively. The patients in the severe group were older than other groups (P<0.005) and tended to be male (p = 0.07). The median onset of symptoms in this study was 5 days (IQR 2-11days). The median onset of symptoms after exposure was 8 days (IQR 5–11 days).

#### Testing of archived negative control and PCR-confirmed COVID-19 patient samples by sVNT

Two hundred ninety-eight blood samples from negative control group (healthy and negative COVID-19 PCR testing in NT) were tested by sVNT. All samples were negative for SARS-CoV-2 neutralizing antibodies as expected, yielding an overall specificity of 100% (95% confidence interval (CI) 98.3–100%) (S1 Table).

We then applied sVNT to samples from 97 patients with PCR-confirmed COVID-19 whose blood specimens were available for sVNT assay. The positive predictive value (PPV) was 100% (95% CI 96.2–100%). The sensitivity was 99.0% (95% CI 94.4–100%) and negative predictive value (NPV) was 99.7% (95% CI 98.2–100%). One PCR-positive patient, whose blood was collected 4 days after symptom onset, was negative for sVNT (18.8% inhibition) but was positive for IgM ELISA antibody using the same RBD antigen.

The antibody response was dependent on the time post infection and duration after onset of symptoms. This study compared the PPV and NPV results before and day 7 post symptom onset in PCR-confirmed COVID-19 patients. The NPV of sVNT from the samples collected before and after 7 days of symptom onset was 99.7% (95% CI 98.8–100%) and 100% (95% CI 98.8–100%), respectively. The sensitivity and PPV of specimens collected after day 7 of symptom onset was 100% (95% CI 93.8–100%), and before day 7 of symptom onset was 98.8% (95% CI 93.4–100%) and 100% (95% CI 95.6–100%), respectively (S1 Table).

The specificity of sVNT was comparable to the cVNT using live virus. Five serum samples whose titer was determined by cVNT (1:160) were re-tested with sVNT. All five samples showed a good correlation between sVNT and cVNT (S4 Table).

The cutoff %inhibition was 20% as recommended by the manufacturer (GenScript, USA). The median inhibition by sVNT in 258 serum specimens from 97 PCR-confirmed COVID-19 patients was 87.1% (IQR 65.0–92.7), while the median inhibition in negative control samples was 5.0 (IQR 2.6–9.5%, p <0.001), i.e. expectedly lower than the cutoff %inhibition (S1 Fig).

Three serum specimens from PCR-confirmed COVID-19 patients showed less than 20% inhibition with sVNT. The samples from two of these were collected on day 1 after symptom onset and showed 19.2% and 11.5% inhibitions, respectively. Both patients subsequently developed neutralizing antibodies with >20% sVNT inhibition on day 3 after symptom onset. The first serum of the third patient was collected on day 4 after symptom onset and showed 18.8% inhibition. No further blood sample was collected from this patient. Thus, this was the only patient with a negative result within the first 7 days.

In addition, we analyzed the % inhibition by sVNT in patients with low CT value from RT-PCR. All these patients had CT value greater than 30 and 36. The results of sVNT showed that all these patients had the level greater than 20% (S5 Table).

#### Comparison of IgA and IgG (EUROIMMUN) and NAbs (sVNT) detection

**Fig 1:**
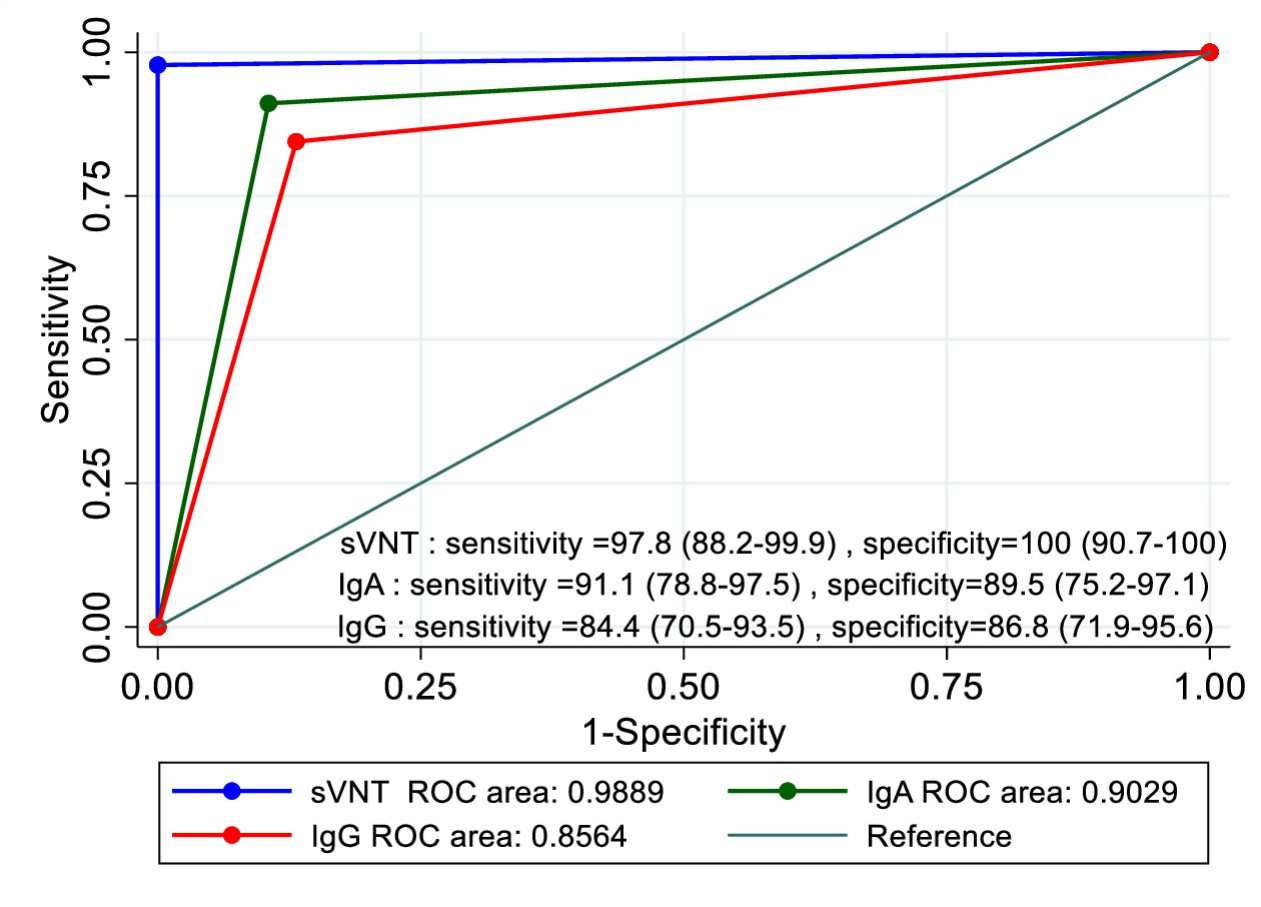
Comparison of Area under the ROC curve (AUC) between sVNT, IgA and IgG tests (PCR-confirmed COVID-19 patients = 45, negative control serum = 38). The correlation is modest between the level of %inhibition by sVNT and the ratio of semi-quantitative measurement of IgA and IgG (R-squared (r2) = 0.386 and 0.343, respectively) by Random-effects generalized least-squares regression.

A total of 88 serum specimens from 45 PCR-positive COVID-19 patients were used to compare the sensitivity between EUROIMMUN ELISAs (IgA and IgG) and sVNT. The sensitivity, specificity, and the area under the receiver operating characteristic (ROC) curve (AUC) was evaluated (Fig 1). The AUC of the sVNT (0.99) was significantly higher than IgA (0.90) and IgG (0.86) (p = 0.005). The sVNT showed highest sensitivity and specificity among the three tests. Twenty-four patients who had negative IgG result but positive sVNT had median %inhibition of 66.6% (IQR 42.3–87.8%), while 6 of 24 were IgA positive. It should be noted that sVNT measures the total antibodies (IgG, IgA and IgM), thus making it a more sensitive test than measuring any single antibody isotype (9).

#### Kinetics of neutralizing antibody compared to number of days after symptom onset

**Fig 2:**
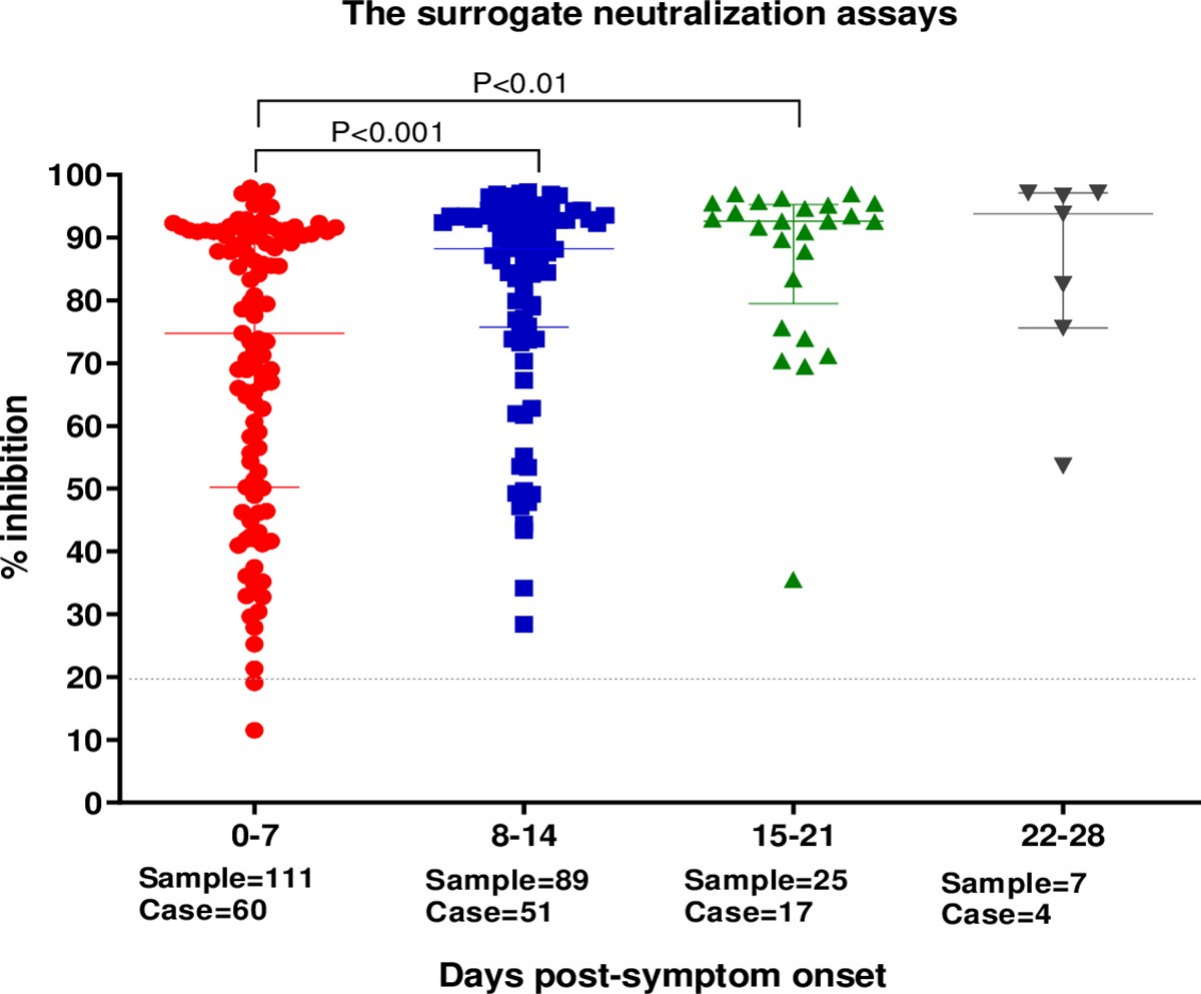
Comparison of sVNT results (%inhibition) between COVID-19 patients on different days after symptom onset. The serology test results of 0–7, 8–14, 15–21, 22–28 days after symptom onset were compared (lateral axis), respectively. Each dot represents one tested specimen and the vertical axis represents the %inhibition of SARS-CoV-2 NAbs tested by sVNT. The dash line represents the cutoff at 20% inhibition. The horizontal lines indicates the median interquartile range (IQR). The p values were calculated from two-tailed test.

Serum specimens (n = 230) from PCR-confirmed COVID-19 patients (n = 71) with more than one blood collection during hospitalization were used to study the antibody response kinetic at different time points. Median percentages of sVNT inhibition increased with time after symptom onset (Fig 2). Nineteen patients’ samples showed sVNT inhibition on day 1 after symptom onset, with median inhibition of 62.0% (IQR; 44.2–96.2). Overall, median inhibition values following symptom onset were: days 0–7, 76% (IQR 51.1–91.2); days 8–14, 88.6% (IQR 76.9–93.5); days 15–21, 91.6% (IQR 73.9–95.1); days 22–28, 93.6% (IQR 82.5–97.0), respectively (Fig 2). The level of inhibition at days 8–14 were significantly higher than days 0–7 (p<0.001).

#### Level of neutralizing antibodies and severity

**Fig 3:**
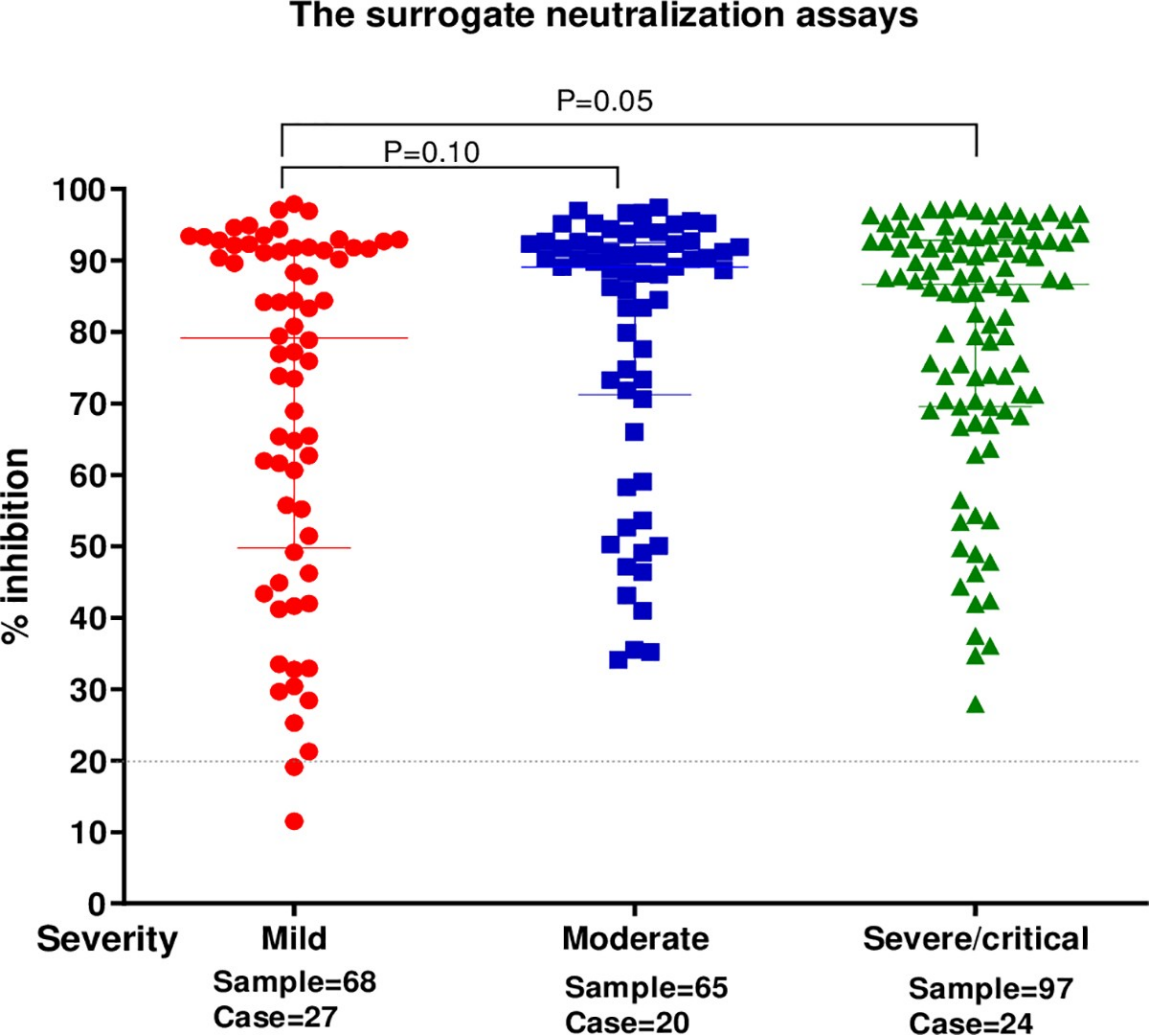
Comparison of sVNT results (%inhibition) between COVID-19 patients with varying degrees of disease severity (mild–asymptomatic or upper respiratory tract infection; moderate–pneumonia without oxygen desaturation, treated with antiviral agents for 5 days or other combined regimens; severe–pneumonia treated with oxygen support and antiviral agents for at least 10 days; critical–pneumonia requiring mechanical ventilation) (lateral axis). Every dot represents one tested specimen and the vertical axis represents the %inhibition of SARS-CoV-2 NAbs tested by sVNT. The dashed line represents the cutoff at 20% inhibition. The horizontal lines indicate the median interquartile range (IQR). The p values were calculated from two-tailed test.

The median levels of sVNT inhibition varied by disease severity (Fig 3). Percentage inhibitions by severity were: mild, 79.9% (IQR 49.7–91.8%), moderate, 89.0% (IQR 71.2–92.4%); severe + critical 86.6% (IQR 69.5–92.8%), respectively. The median level of sVNT %inhibition of severe and critical patients were higher than patients with mild symptoms (p = 0.05) when comparing without distinguishing time points. The median levels of sVNT %inhibition were not significantly different when comparing mild and moderate groups nor when comparing severe and critical with moderate groups (p = 0.1 and p = 0.89, respectively).

The Nabs generally increased with time following symptom onset. Antibody response was significantly higher in the second week after symptom onset compared to the first week (day 0–7) in all disease severity groups (p<0.01) except in moderate group (p = 0.4). Median percentage of inhibition was higher than 90% in the third week after symptom onset in all four disease severity groups (S2 Table). There was no statistical significance in the median %inhibition of sVNT between the 4 severity groups when compared at each time point (p value >0.05).

Notably, in one patient with moderate symptoms, the %inhibition of sVNT were almost constant from symptom onset (50.26% and 50.08% inhibition at days 6 and 7 after symptom onset, respectively to when they were discharged on day 24 (53.63% inhibition)). This could represent an unusual case of slow kinetics of Nab development where the peak level was yet to be achieved. We were unable to obtain further sequential samples from the patient, thus further investigation of this unusual case was not possible.

#### Neutralizing antibody and patient age and sex

**Fig 4:**
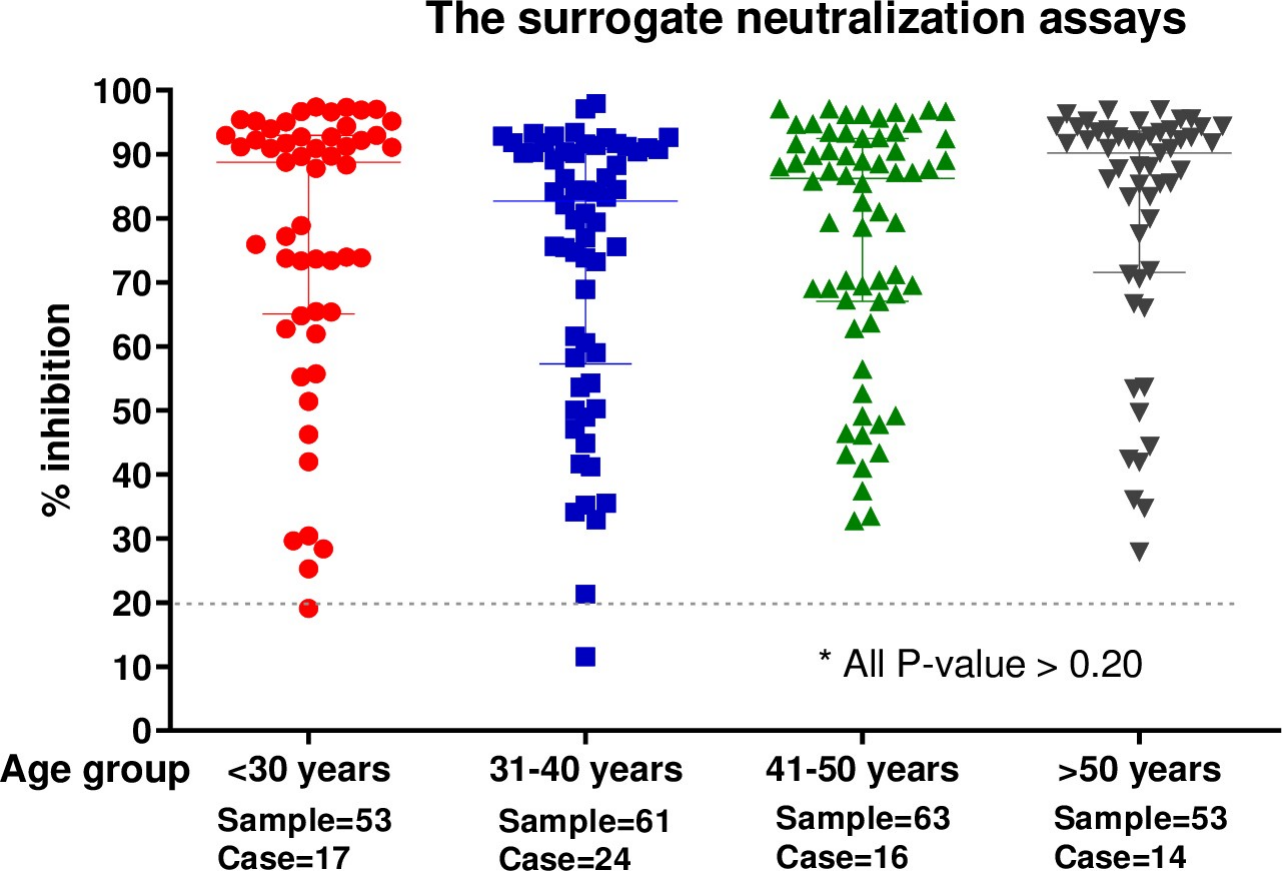
Comparison of sVNT results (%inhibition) between COVID-19 patients in different age groups (lateral axis). Every dot represents one tested specimen and the vertical axis represents the %inhibition of SARS-CoV-2 NAbs tested by sVNT. The dashed line represents the cutoff at 20% inhibition. The horizontal lines indicate the median interquartile range (IQR). The p values were calculated from two-tailed test.

There was no difference in the median %inhibition of sVNT by age or gender groupings. Median %inhibition by age group (years) was: age 18–30, 88.8% (IQR 65.4–92.9%); 31–40, 83.3% (IQR 58.3–90.8%); 41–50, 85.8% (IQR67-92.4%); >50, 90.2% (IQR 71.8–93.5%) (p = 0.58) (Fig 4). Median %inhibition by gender was 87.8% (95% IQR 58.3–92.7%) and 86.2% (95% IQR 70.6–91.6%), for female and males, respectively (S3 Table).

## Discussion

This study was conducted in an Emerging Infectious Diseases Centre which served patients with SARS-CoV-2 infection. Most of the patients in this study presented with mild infections however, 18% presented as severe or critical. Asymptomatic infection was not included in our study. Most patients in this study presented for screening of COVID-19 at relatively early stage of infection due to an extensive contact tracing from two major super spreader events in Bangkok. Most patients with severe infections were male and elderly patients, which is concordant with previous studies [10–13]. This study evaluated the efficacy of sVNT as a means to early detect NAbs against SARS-CoV-2 in patient blood samples.

In our study, commercially available sVNT assay, which was the same platform as previously published [9], was used to determine the levels of neutralizing antibodies from PCR-confirmed COVID-19 patients from our cohort in Thailand. The overall sensitivity of sVNT in our study was 99.0% (n = 97 patients); 98.8% during days 0–7, and 100% 7 days after onset of symptoms. These findings corroborate with the published findings by Tan and colleagues [9] and demonstrate the efficacy of the assay to detect antibodies against SARS-CoV-2 within the first week of symptom onset.

Of COVID-19 patients in our study, 81 of 82 (98.8%) were sVNT positive during the first week after symptom onset, demonstrating the possibility of using sVNT an adjunct tool for detection of COVID-19 infection together with the standard rRT-PCR. In most patients with serial measurements of sVNT, the levels of %inhibition rapidly increased at the beginning of second week after symptom onset and peaked in the third week after symptom onset. In a different study, 43% of the patients had detectable cVNT in the first week upon onset of symptoms and 100% at days 21–28 [14]. From this study, sVNT has shown higher sensitivity than cVNT during the first week after symptom onset. The longest duration of detectable NAbs by sVNT in our study was 32 days after onset of symptoms. NAb levels over a longer period should be monitored to determine the kinetics of antibody response months after infection.

The overall sensitivity of sVNT, IgA, and IgG antibodies using the same group of specimens were 97.8%, 91.1%, and 84.4%, respectively (Fig 4). The results suggest that sVNT is more sensitive and able to detect positive antibody responses earlier than the IgA or IgG ELISA against the S antigen. This result was in accordance to previous studies [9, 14, 15] and revealed that the total antibody sVNT assay is a better tool for antibody detection than IgA or IgG alone. To explore the kinetics of IgA, IgG and sVNT, time at the starting point is needed to be adjusted.

A large meta-analysis by Deeks and colleagues examined 54 serology-related publications analyzing over 15,000 samples, and found that combined IgM/IgG assays showed sensitivities over all time points ranged from 97.2% to 99.4% [16]. Our assay does not specify antibody type, but rather quantifies total neutralization antibodies directly in one single test and can yield such information without the need to perform PRNT.

Additionally, the specificity of sVNT in multiple samples obtained from various clinical settings collected during the SARS-CoV-2 pandemic was 100%. All 298 serum specimens showed less than 20% inhibition (manufacturer’s recommended cutoff criteria). The cutoff for %inhibition at 20% in this study showed good sensitivity and specificity. However, the cutoff level could be adjusted in each country before use as a routine diagnostic assay as appropriate. It was shown in previous studies that the sVNT assays can differentiate antibody responses to SARS-CoV-2 from seasonal human CoV infections, but showed cross-reaction with archived serum of SARS patients [16, 17]. Our data strongly suggested that sVNT is a useful serological test for SARS-CoV-2 infection. It is important to point out that, to date, cPass^TM^ is the only commercially available serological test which directly measures NAbs against SARS-CoV-2. While the relationship between the protective immunity and NAbs level is yet to be conclusively demonstrated for SARS-CoV-2, it is worth noting that almost all published studies of vaccine development have used NAbs as one of the key performance indicators for a successful protective immune response in humans or animals [18, 19]. In addition, a recent study of a cohort on a fishing boat, using the same cPass^TM^ assay as ours, provided strong evidence to suggest that NAbs play a key role in protective immunity [8, 20].

By measuring %inhibition of sVNT in blood samples of patients with varying degrees of COVID-19 severity, it was observed that levels of NAbs in severe and critical groups were significantly higher than the mild group (p = 0.05). This finding is also analogous to other studies that measured NAbs in COVID-19 patients [7, 9, 15, 21, 22]. There was no significant difference in %inhibition between mild and moderate (p = 0.1) or severe and moderate groups (p = 0.89). This finding is again similar to published study in which no significant difference in the kinetics, magnitude, or functionality of the response between hospitalized patients at general ward or the ICU was found [13]. The relationship between NAb levels and disease severity has yet to be further clarified [3, 17].

The median level of %inhibition of sVNT during the first week of symptom onset in the mild group in our study were higher than in the study conducted in the Caucasian population with mostly mild disease [23]. The difference in the %inhibition from these two studies may be derived from difference in population, host genetics and viral variants.

The limitations of this study are the relatively short duration of follow-up time and the fact that blood specimens were not collected on the same day after symptom onset in all patients for direct comparison. The level of long-term persistence of NAbs requires further studies and a larger sample size.

## Conclusion

Our findings suggest that sVNT is a practical and robust serological test for SARS-CoV-2 infection. This assay provides accurate and timely results and can be conducted in most BSL-2 laboratory settings without requirement of high-level biosafety containment. It can be used to determine the protective immune response from SARS-CoV-2 infection.

## Supporting information

S1 Fig The % inhibition by sVNT of PCR-confirmed SARS-CoV-2 infection, samples from prepandemic era and other infection. (DOCX)

S2 Fig The % inhibition by sVNT and days after symptom of onset. (DOCX)

S1 Table Sensitivity, specificity, PPV and NVP of sVNT for diagnosis of SARS-CoV-2 infection. (DOCX)

S2 Table Median (IQR) % inhibition of sVNT by severity group. (DOCX)

S3 Table Median (IQR) % inhibition of sVNT by sex. (DOCX)

S4 Table The correlation between sVNT and cVNT. (DOCX)

S5 Table The level of % inhibition from sVNT in participants with CT value from RT-PCR > 30 and > 36. (DOCX)

S1 Dataset (XLSX)
