# Supplementary material for: Early detection of neutralizing antibodies against SARS-CoV-2 in COVID-19 patients in Thailand
Source: PLoS One. 2021 Feb 12;16(2):e0246864. doi: 10.1371/journal.pone.0246864 (PMC7880427; doi:10.1371/journal.pone.0246864)
Supplement: S1 Fig — (DOCX) [file pone.0246864.s001.docx]

**S1 Fig. The % inhibition by sVNT of PCR-confirmed SARS-CoV-2 infection, samples from prepandemic era and other infection.**
