# Supplementary material for: Early detection of neutralizing antibodies against SARS-CoV-2 in COVID-19 patients in Thailand
Source: PLoS One. 2021 Feb 12;16(2):e0246864. doi: 10.1371/journal.pone.0246864 (PMC7880427; doi:10.1371/journal.pone.0246864)
Supplement: S1 Table — (DOCX) [file pone.0246864.s003.docx]

**S1 Table. Sensitivity, specificity, PPV and NVP of sVNT for diagnosis of SARS-CoV-2 infection.**

|  | All | | Day 0-7 | | Day >7 | |
| --- | --- | --- | --- | --- | --- | --- |
|  | n/N | % (95%CI) | n/N | % (95%CI) | n/N | % (95%CI) |
| Sensitivity  (positive/total) | 96/97 | 99.0 (94.4-100) | 81/82 | 98.8 (93.4-100) | 58/58 | 100 (93.8-100) |
| Specificity  (negative/total) | 212/212 | 100 (98.3-100) | 212/212 | 100 (98.3-100) | 212/212 | 100 (98.3-100) |
| PPV  (true positive/patient) | 96/96 | 100 (96.2-100) | 82/82 | 100 (95.6-100) | 58/58 | 100 (93.8-100) |
| NPV  (true neg/total neg) | 298/299 | 99.5 (97.4-100) | 212/213 | 99.5 (97.4-100) | 212/212 | 100 (98.3-100) |

N: number of total patient tested, n: number of positive/negative patient
