# Supplementary material for: Early detection of neutralizing antibodies against SARS-CoV-2 in COVID-19 patients in Thailand
Source: PLoS One. 2021 Feb 12;16(2):e0246864. doi: 10.1371/journal.pone.0246864 (PMC7880427; doi:10.1371/journal.pone.0246864)
Supplement: S2 Table — (DOCX) [file pone.0246864.s004.docx]

**S2 Table. Median (IQR) % inhibition of sVNT by severity group.**

| Day after symptom of onset | Mild | Moderate | Severe | Critical | P-value |
| --- | --- | --- | --- | --- | --- |
| 0-7 | N=41 | N=34 | N=27 | N=9 |  |
|  | 65.5 (41.7-90.2) | 87.5 (59-91.2) | 78.6 (46.2-88.9) | 69 (63.6-90.9) | 0.38 |
| 8-14 | N=23 | N=25 | N=26 | N=15 |  |
|  | 84.4 (61.9-92.7) | 90.2 (84.5-94.3) | 89 (70.3-94.4) | 87.1 (82.1-92.9) | 0.60 |
| 15-21 | N=4 | N=5 | N=11 | N=5 |  |
|  | 93.8 (91.3-95.8) | 90.9 (83.4-94) | 92.6 (71.2-95.7) | 92.5 (87.7-92.6) | 0.97 |

P-value for compare P% of surrogate neutralization assays at each days after symptom onset between severity group using Generalized estimating equations (GEE) with linear model

N = number of tested specimen
