# Supplementary material for: Early detection of neutralizing antibodies against SARS-CoV-2 in COVID-19 patients in Thailand
Source: PLoS One. 2021 Feb 12;16(2):e0246864. doi: 10.1371/journal.pone.0246864 (PMC7880427; doi:10.1371/journal.pone.0246864)
Supplement: S3 Table — (DOCX) [file pone.0246864.s005.docx]

**S3 Table. Median (IQR) % inhibition of sVNT by sex.**

|  | Female | Male | P-value |
| --- | --- | --- | --- |
| 0-7 | N=65 | N=46 |  |
|  | 74.8 (46.4-91.2) | 75.7 (55.7-90.1) | 0.86 |
| 8-14 | N=43 | N=46 |  |
|  | 92.3 (70.3-94.3) | 86.4 (77.2-91.8) | 0.92 |
| 15-21 | N=10 | N=15 |  |
|  | 92.7 (71.2-95.2) | 92.6 (83.4-95.5) | 0.46 |
| 22-28 | N=1 | N=4 |  |
|  | 53.6 | 95.2 (88.2-96.9) | <0.001 |

P-value for compare P% of surrogate neutralization assays at each days after symptom onset between sex using Generalized estimating equations (GEE) with linear model

N = number of specimens
