# Supplementary material for: Early detection of neutralizing antibodies against SARS-CoV-2 in COVID-19 patients in Thailand
Source: PLoS One. 2021 Feb 12;16(2):e0246864. doi: 10.1371/journal.pone.0246864 (PMC7880427; doi:10.1371/journal.pone.0246864)
Supplement: S4 Table — (DOCX) [file pone.0246864.s006.docx]

**S4 Table. The correlation between sVNT and cVNT.**

| MID | Specimen No. | Dilution | NT Result | OD | Control | % inhibition by sVNT |
| --- | --- | --- | --- | --- | --- | --- |
| SI2015257-SR1 | CCP.63.0.00039 | 1:10 | Positive | 0.1017 | 1.6553 | 94 |
| SI2015257-SR2 | CCP.63.0.00039 | 1:20 | Positive | 0.7249 | 1.6553 | 56 |
| SI2015257-SR3 | CCP.63.0.00039 | 1:40 | Positive | 1.1865 | 1.6553 | 28 |
| SI2015257-SR4 | CCP.63.0.00039 | 1:80 | Positive | 1.2212 | 1.6553 | 26 |
| SI2015257-SR5 | CCP.63.0.00039 | 1:160 | Positive | 1.2508 | 1.6553 | 24 |
| SI2015257-SR6 | CCP.63.0.00039 | 1:320 | Negative | 1.6728 | 1.6553 | 0 |
| SI2015258-SR1 | CCP.63.0.00040 | 1:10 | Positive | 0.2624 | 1.6553 | 84 |
| SI2015258-SR2 | CCP.63.0.00040 | 1:20 | Positive | 0.5287 | 1.6553 | 68 |
| SI2015258-SR3 | CCP.63.0.00040 | 1:40 | Positive | 0.6259 | 1.6553 | 62 |
| SI2015258-SR4 | CCP.63.0.00040 | 1:80 | Positive | 1.1971 | 1.6553 | 28 |
| SI2015258-SR5 | CCP.63.0.00040 | 1:160 | Positive | 1.2302 | 1.6553 | 26 |
| SI2015258-SR6 | CCP.63.0.00040 | 1:320 | Negative | 1.6521 | 1.6553 | 0 |
| SI2015259-SR1 | CCP.63.0.00041 | 1:10 | Positive | 0.2703 | 1.6553 | 84 |
| SI2015259-SR2 | CCP.63.0.00041 | 1:20 | Positive | 0.7841 | 1.6553 | 53 |
| SI2015259-SR3 | CCP.63.0.00041 | 1:40 | Positive | 1.0766 | 1.6553 | 35 |
| SI2015259-SR4 | CCP.63.0.00041 | 1:80 | Positive | 1.1694 | 1.6553 | 29 |
| SI2015259-SR5 | CCP.63.0.00041 | 1:160 | Positive | 1.251 | 1.6553 | 24 |
| SI2015259-SR6 | CCP.63.0.00041 | 1:320 | Negative | 1.5922 | 1.6553 | 4 |
| SI2015260-SR1 | CCP.63.0.00053 | 1:10 | Positive | 0.5764 | 1.6553 | 65 |
| SI2015260-SR2 | CCP.63.0.00053 | 1:20 | Positive | 0.8691 | 1.6553 | 47 |
| SI2015260-SR3 | CCP.63.0.00053 | 1:40 | Positive | 1.0427 | 1.6553 | 37 |
| SI2015260-SR4 | CCP.63.0.00053 | 1:80 | Positive | 1.183 | 1.6553 | 29 |
| SI2015260-SR5 | CCP.63.0.00053 | 1:160 | Positive | 1.2269 | 1.6553 | 26 |
| SI2015260-SR6 | CCP.63.0.00053 | 1:320 | Negative | 1.517 | 1.6553 | 8 |
| SI2015261-SR1 | CCP.63.0.00068 | 1:10 | Positive | 0.5818 | 1.6553 | 65 |
| SI2015261-SR2 | CCP.63.0.00068 | 1:20 | Positive | 0.8418 | 1.6553 | 49 |
| SI2015261-SR3 | CCP.63.0.00068 | 1:40 | Positive | 1.1063 | 1.6553 | 33 |
| SI2015261-SR4 | CCP.63.0.00068 | 1:80 | Positive | 1.1252 | 1.6553 | 32 |
| SI2015261-SR5 | CCP.63.0.00068 | 1:160 | Positive | 1.2311 | 1.6553 | 26 |
| SI2015261-SR6 | CCP.63.0.00068 | 1:320 | Negative | 1.3328 | 1.6553 | 19 |
