# Supplementary material for: Early detection of neutralizing antibodies against SARS-CoV-2 in COVID-19 patients in Thailand
Source: PLoS One. 2021 Feb 12;16(2):e0246864. doi: 10.1371/journal.pone.0246864 (PMC7880427; doi:10.1371/journal.pone.0246864)
Supplement: S5 Table — (DOCX) [file pone.0246864.s007.docx]

**S5 Table. The level of % inhibition from sVNT in participants with CT value from RT-PCR > 30 and > 36.**

| **PID** | **Age** | **CT** | **%sVNT** |
| --- | --- | --- | --- |
| **CT from RT-PCR >30** | | | |
| **336233/57** | **52** | **31.68** | **91.88** |
| **28574/63** | **23** | **33** | **97.39** |
| **21965/62** | **36** | **33** | **91.50** |
| **60704/60** | **38** | **34.88** | **97.92** |
| **29255/63** | **20** | **35.19** | **96.83** |
| **72400/54** | **35** | **34.89** | **90.72** |
| **30220/63** | **36** | **36.29** | **50.26** |
| **30417/63** | **31** | **34.63** | **91.23** |
| **25701/63** | **26** | **33.18** | **92.73** |
| **31666/63** | **43** | **30.7** | **37.46** |
| **111827/55** | **38** | **31.1** | **59.04** |
| **26120/63** | **37** | **36.26** | **21.30** |
| **CT from RT-PCR > 36** | | | |
| **30220/63** | **36** | **36.29** | **50.26** |
| **26120/63** | **37** | **36.26** | **21.30** |
